# Patterns of Sexual Behavior in Lowland Thai Youth and Ethnic Minorities Attending High School in Rural Chiang Mai, Thailand

**DOI:** 10.1371/journal.pone.0165866

**Published:** 2016-12-01

**Authors:** Linda Aurpibul, Arunrat Tangmunkongvorakul, Patou Masika Musumari, Kriengkrai Srithanaviboonchai, Surapee Tarnkehard

**Affiliations:** 1 Research Institute for Health Sciences, Chiang Mai University, Chiang Mai, Thailand; 2 Department of Global Health and Socio-epidemiology, Kyoto University School of Public Health, Kyoto, Japan; 3 Faculty of Medicine, Chiang Mai University, Chiang Mai, Thailand; University of Westminster, UNITED KINGDOM

## Abstract

**Introduction:**

The rural areas of Northern Thailand are home to a large cultural diversity of ethnic minority groups. Previous studies have shown that young people in rural Thailand have low levels of knowledge on HIV/AIDS and high sexual risks. We compared sexual behaviors between the lowland Thai youth and the youth from ethnic minority groups.

**Methods and findings:**

This is a cross-sectional quantitative study conducted among high-school Thai and ethnic students in Chiang Mai. From a total 1215 participants, 487 (40.1%) were lowland Thai and 728 (59.9%) were from ethnic minorities. Overall, 17.9% of respondents reported “ever had sex.” Lowland Thai adolescents were more likely to have ever had sex compared with ethnic minority adolescents (AOR, 1.61; CI, 1.06–2.45; *P*< 0.01). A higher proportion of lowland Thai respondents reported having ≥ 2 lifetime sexual partners (51.9% vs. 33.3%, *P* = 0.003), or currently having a boy/girlfriend (59.9% vs. 45.3%, *P*< 0.001) compared to ethnic minority adolescents. Consistent condom use was low in both groups (22.6%). The common significant factors associated with "ever had sex" in both groups were "ever drunk alcohol in the past year" and "currently having a boy/girlfriend." Specifically, for lowland Thai youth, being around the age of 17 or 18 years and "ever used methamphetamine in the past year" were associated with increased odds of “ever had sex”. For ethnic minority adolescents, being female and belonging to religions other than Buddhism were associated with decreased odds of “ever had sex”.

**Conclusion:**

A substantially higher proportion of lowland Thai engage in risky sexual behaviors when compared to ethnic minorities. However, both groups remained vulnerable to HIV and other sexually transmitted infections. To minimize sexual risks, education program and school-based interventions are warranted to increase awareness of young people about risky behaviors and to promote essential life skills.

## Introduction

Risky sexual behaviors continue to raise the HIV epidemic among young people [1, 2]. In 2010, young people aged 15–24 years accounted for 42% of all new global HIV infections in people aged 15 years and older [3]. As described in detail elsewhere [4–9], sexual norms and traditional values of young people in Thailand are now undergoing rapid transformations- fueled by globalization and also by the increasing urbanization that the country has been witnessing over the past decades. These changes in sexual norms which include a decline in the age of sexual onset both in male and female Thai youth, a larger number of lifetime sexual partners, and a greater degree of acceptance of adolescent premarital sex [5], are also reflected in the increasing rates of unintended pregnancies and sexually transmitted infections (STIs) among Thai adolescents over the past 15 years [8, 9].

Although adolescents are identified as one of the most-at risk population groups in Thailand, it is important to note that they make up a heterogeneous population with different degrees of risk and vulnerability profiles [10]. Our recent study conducted in urban Chiang Mai revealed that a high proportion of young people enrolled in non-formal education centers engaged in risky sexual behavior, notably low condom use and high number of lifetime sexual partners [11]. This is consistent with results from our earlier work showing that a higher proportion of out-of-school Thai youth (One third was sampled from non-formal education centers) engaged in risky sexual behavior compared to those enrolled in general school and university [4, 12, 13].

However, the patterns of sexual behavior of other specific sub-groups of adolescents are still not well captured in the existing literature. These adolescents particularly include lowland Thai and ethnic minorities occupying rural areas of the Northern Thailand, including Chiang Mai province. The rural Northern Thailand, mainly occupied by the lowland Thai who are the majority ethnic group in the lowland areas, is also home to a large number of diversified ethnic minority groups. The main groups include the Hmong, Karen, Akha, Lisu, Shan, and Yunnanese from China.

The Shan people are a migrant group who reside mainly in the lowland rural areas of Chiang Mai province. They have migrated across the Thai-Burmese border from Shan state in the mid-Eastern part of Myanmar, and are mostly employed in the agricultural and industrial sectors [14]. The other ethnic minority groups mostly occupy the higher mountainous areas of the Northern region; thus, they are generally referred to as hill tribes. They migrated from the Asian interior over a century ago. Most ethnic minority groups have in common the fact that they have lived for long periods of time in cultural and geographic isolation; mostly have practiced subsistence farming and agriculture; and have been largely socio-economically worse-off when compared to lowland Thai [15].

Many of the ethnic minority groups have been converted to Christianity by the missionaries, who have also provided diverse socio-economic support to improve their standard of living [16]. Currently, many young people from ethnic minority groups seek work opportunities in the lowland where they account for a sizeable proportion of the unskilled labor force. They also have access to further educational opportunities through the governmental scheme promoting free access to primary education at the district level, as well as through other non-governmental organizations including Christian groups which support young from ethnic minorities to pursue higher education [17]. The members of the Yunnan ethnic group whose ancestors migrated overland from Yunnan province in southwestern China are not officially recognized as a hill tribe by the Thai government. However, they dwell in the same geographic location as the other upland hill tribes, with whom they share common challenges and socio-economic conditions [18].

Thus, lowland Thai youth plus youth from the aforementioned ethnic minority groups make up the bulk of adolescents attending high-school in rural Chiang Mai. Adolescents in rural Chiang Mai Province and in rural areas of northern Thailand at large may disproportionately be vulnerable to HIV infection and other STIs. The vulnerability is partly due to their limited access to sexual and reproductive health education and to health services in rural settings, which are mainly administered by various government sectors [19]. Risky patterns of sexual behavior among adolescents in this region remain poorly documented, and our previous work mainly focused on adolescents in urban Chiang Mai. A few studies have explored social and cultural factors leading to sexual risk behaviors, or attitudes and behavior regarding sexual intercourse [20, 21]. However, none of them has identified the correlates of risky sexual behaviors or explored whether there exist any similarities and differences in the nature of these correlates between lowland Thai and ethnic minority groups in rural Northern, Thailand. In an attempt to address the above-cited gaps in research, the current study aims to identify correlates of sexual intercourse among lowland Thai and ethnic minority groups attending high school in rural Chiang Mai, Thailand.

## Methods

### Ethics statement

The study was approved by the Human Experimentation Committee of the Office of Research Ethics of the Research Institute for Health Sciences, Chiang Mai University (Certificate approval number 52/2014). Before participating, potential participants were first informed about the study’s objectives; about their roles and their rights to give or not to give any information during the interview; about the confidentiality of the personal data; and about the way that the findings of the study would be presented.

Participants provided verbal informed consent prior to participating in the study. The verbal informed consent was preferred to the written consent, based on the vulnerable nature of our study population; and also in order to prevent potential harm that could result from a breach of confidentiality. The process of informed consent was deemed appropriate by the Office of Research Ethics. For participants under 18 years old, written informed consent was obtained from their guardians- after providing them with all the information regarding the study.

### Study design, population, & setting

This is a cross-sectional quantitative study which was conducted in rural Chiang Mai Province between July and December 2014. Prospective respondents were recruited from among high school students studying in grades 11–12. They were recruited from all the 8 main government secondary schools of 5 districts—including Samoeng, Phrao, Chiang Dao, Mae Ai, and Mae Chaem. All 5 districts are in mountainous areas, located more than 60 kilometers from Chiang Mai City.

The study sample was categorized into 2 groups, including 1) lowland Thai (defined as youth with Thai citizen who were born to Thai parents), and 2) ethnic minorities (Hmong, Karen, Akha, Lisu, Shan, and Yunnanese from China), according to their self-identification of their ethnicity when completing the questionnaire. All students in Grades 11 and 12 were invited to participate. They received the study information from the study staff and had a chance to review the information sheet before decided whether to participate in the study. The questionnaires were prepared in Thai language, and students were asked to complete them by themselves after being orientated by study staff. All respondents, including the ethnic minorities, have been studying in Thai government schools using Thai language in classroom for more than 10 years so both group were comparably proficient in Thai language in which the questions were asked. Data collection occurred on school grounds; however, neither teachers nor any staff members affiliated with the school were present while students were completing the questionnaire. All data collected was confidential and identified by code numbers only.

### Data collection, instruments, & variables

A self-administered questionnaire was used to collect the data (S1 and S2 Questionnaires). The questionnaire covered socio-economic background; recreational activities; alcohol, tobacco and drug use; relationships; sexual experience; sexually transmitted diseases; birth control; pregnancy and abortion; and degree of need for sexual health services. A field research team received training on questionnaire administration and was appropriately informed on the general and specific objectives of the study.

The selected main outcome of this study was “ever had sexual intercourse”. The first set of covariates was made up of socio-economic variables: age; sex; religion; living situation; and degree of access to mobile phone or Internet. The second set of covariates related to substance use: alcohol drinking; smoking; and drug use. The third set of covariates was made up of sexual behavior-related factors: currently having a boyfriend/girlfriend; sexual history and experience; age of sexual debut; use of condoms and contraception; sexually transmitted diseases; and pregnancy.

### Data analysis

The analysis was conducted using SPSS (PASW) for Windows 17.0 (SPSS Inc., Chicago, Illinois, USA) (S1 Dataset). Univariate analysis was used to obtain descriptive statistics of the sample. Chi-square tests for categorical variables and student t-tests for continuous variables were used to compare lowland Thai youth and ethnic minority group youth with respect to socio-demographic factors; substance use factors; and sexual behavior-related factors. We also aimed to document the correlates of "ever had sexual intercourse," comparing lowland Thai and ethnic minority groups. In this regard, we used multiple logistic regressions to obtain adjusted odds ratios (AOR) and 95% confidence intervals (CI) of factors associated with “ever had sexual intercourse.” Only variables that were significant (*P*<0.05) or those that were judged epidemiologically important were included in the multivariate models. The diagnostic procedures yielded no evidence of multicollinearity.

## Results

### Socio-demographic information

A total of 1,215 participants were contacted; all completed the study. The mean age [standard deviation (SD)] of respondents was 17.27 (0.76) years. The majority of participants were female (68.1%); aged less than 18 years (66.6%); Buddhist by religion (73.5%); lived with parents or relatives (64%); either had a mobile phone themselves or could access one their family owned (98.3%); and had access to internet (owned one themselves, or had access to one their family owned) (55.5%).

In terms of the ethnicities of the participants, 487 (40.1%) were lowland Thai (172 males and 315 females), while 728 (59.9%) were from ethnic minorities (215 males and 513 females). The mean (SD) age of lowland Thai youth were lower when compared to the youth from ethnic minorities [17.12 (0.60) vs. 17.37 (0.84), p< 0.001). While most of the lowland Thai youth were Buddhist (95.9%), a significant proportion of the ethnic minority youth were Christian (41.2%). The living status was also different between the two groups. The majority of lowland Thai respondents (82.3%) lived with parents or relatives; only a few lived with teachers (9.2%). However, 37.6% of ethnic minorities lived with teachers at the school. When compared to ethnic minorities, a significantly higher proportion of lowland Thai youth owned mobile phones themselves (91.0% vs. 70.7%, p< 0.001), and had access to the Internet at home; and either owned an Internet-connected device or had access to one in the family (59.8% vs. 51.7%, p< 0.001) (see Table 1).

**Table 1:** Socio-demographic characteristics of high-school students in rural Chiang Mai, Thailand.

	Lowland Thai			Ethnic minorities ^1^			*P* value^2^	Total (N = 1215)
	Males (N = 172)	Females (N = 315)	Total (N = 487)	Males (N = 215)	Females (N = 513)	Total (N = 728)		
**Age**								
16	13 (7.6)	38 (12.1)	51 (10.5)	25 (11.6)	81 (15.8)	106 (14.6)	<0.001	157 (12.9)
17	114 (66.3)	222 (70.5)	336 (69.0)	92 (42.8)	224 (43.7)	316 (43.4)		652 (53.7)
18	38 (22.1)	52 (16.5)	90 (18.5)	81 (37.7)	159 (31.0)	240 (33.0)		330 (27.2)
19	7 (4.1)	3 (1.0)	10 (2.1)	17 (7.9)	49 (9.6)	66 (9.1)		76 (6.3)
Mean age (SD)	17.23 (0.64)	17.06 (0.57)	17.12 (0.60)	17.42 (0.80)	17.34 (0.86)	17.37 (0.84)		17.27 (0.76)
**Religion**								
Buddhism	164 (95.3)	303 (96.2)	467 (95.9)	134 (62.3)	292 (56.9)	426 (58.5)	<0.001	893 (73.5)
Christianity	8 (4.7)	11 (3.5)	19 (3.9)	80 (37.2)	220 (42.9)	300 (41.2)		319 (26.3)
Islam	0 (0.0)	1 (0.3)	1 (0.2)	0 (0.0)	0 (0.0)	0 (0.0)		1 (0.1)
Others	0 (0.0)	0 (0.0)	0 (0.0)	1 (0.5)	0 (0.0)	1 (0.1)		1 (0.1)
Missing	0 (0.0)	0 (0.0)	0 (0.0)	0 (0.0)	1 (0.2)	1 (0.1)		1 (0.1)
**Living situation**								
Live with parent(s) or relatives	155 (90.1)	246 (78.1)	401 (82.3)	111 (51.6)	266 (51.9)	377 (51.8)	<0.001	778 (64.0)
Live with teacher(s)	8 (4.7)	37 (11.7)	45 (9.2)	74 (34.4)	200 (39.0)	274 (37.6)		319 (26.3)
Live with friends	0 (0.0)	5 (1.6)	5 (1.0)	9 (4.2)	23 (4.5)	32 (4.4)		37 (3.0)
Live alone	9 (5.2)	26 (8.3)	35 (7.2)	9 (4.2)	11 (2.1)	20 (2.7)		55 (4.5)
Live with other**s**	0 (0.0)	0 (0.0)	0 (0.0)	12 (5.6)	13 (2.5)	25 (3.4)		25 (2.1)
Missing	0 (0.0)	1 (0.3)	1 (0.2)	0 (0.0)	0 (0.0)	0 (0.0)		1 (0.1)
**Have access to mobile phone**								
I own one myself	162 (94.2)	281 (89.2)	443 (91.0)	153 (71.2)	362 (70.6)	515 (70.7)	<0.001	958 (78.8)
I have access to one family own	10 (5.8)	32 (10.2)	42 (8.6)	59 (27.4)	136 (26.5)	195 (26.8)		237 (19.5)
No, I do not have access to one at home	0 (0.0)	0 (0.0)	0 (0.0)	1 (0.5)	4 (0.8)	5 (0.7)		5 (0.4)
Missing	0 (0.0)	2 (0.6)	2 (0.4)	2 (0.9)	11 (2.1)	13 (1.8)		15 (1.2)
**Have access to internet**								
I own one myself	82 (47.7)	176 (55.9)	258 (53.0)	87 (40.5)	202 (39.4)	289 (39.7)	<0.001	547 (45.0)
I have access to one my family owns	8 (4.7)	25 (7.9)	33 (6.8)	30 (14.0)	65 (12.7)	95 (13.0)		128 (10.5)
No, I do not have access to one at home	74 (43.0)	107 (34.0)	181 (37.2)	81 (37.7)	216 (42.1)	297 (40.8)		478 (39.3)
Missing	8 (4.7)	7 (2.2)	15 (3.1)	17 (7.9)	30 (5.8)	47 (6.5)		62 (5.1)

^1^ Ethnic minorities: Hmong, Karen, Akha, Lisu, Shan, Burmese, and Yunnanese

^2^ compared all lowland Thai and all ethnic minorities;

p-value from Chi-square test

*p <0.05

**p <0.01

***p < 0.001

### Alcohol, smoking, and substance use

Nearly half of all respondents reported “ever having drunk alcohol” in the past year (67.4% among males and 32.6% among females). Ninety-one percent drank less than once a week. Eleven percent of all the study participants (27.6% of males and 3.0% of females) reported “ever having smoked” in the past year. Among those who smoked, 65.9% percent smoked occasionally, while 23% smoked 1–5 cigarettes per day. None reported “ever having used heroine or other illegal drugs” (injected or other forms) in the past year. Few reported “ever having used glue or ice drugs” (≤ 1% of males, none of females). Nine percent of females and 5% of males had “ever used marijuana” and “ever used methamphetamine” in the past year, while only a few females reported ever having used them (≤ 0.5%).

When comparing the lowland Thai youth and the youth from the ethnic minority groups, a higher proportion of lowland Thai “had ever drunk alcohol in the past year" (53.8% vs. 37.0%, P< 0.001). Of those who “had ever drunk,” a higher proportion of lowland Thai youth respondents reported drinking ≥ 1 time/week. There was no statistically significant difference between the two groups in terms of smoking; however, a sizeable proportion (7.3%) of ethnic minorities had no data regarding tobacco smoking (see Table 2).

**Table 2:** Alcohol, smoking, and substance use among high-school students in rural Chiang Mai, Thailand.

	Lowland Thai			Ethnic minorities^1^			*P* value^2^	Total		
	Males (N = 172)	Females (N = 315)	Total (N = 487)	Males (N = 215)	Females (N = 513)	Total (N = 728)		Males (N = 387)	Females (N = 828)	Total (N = 1215)
**Ever drunk Alcohol in past year**										
Yes	125 (72.7)	137 (43.5)	262 (53.8)	136 (63.3)	133 (25.9)	269 (37.0)		261 (67.4)	270 (32.6)	531 (43.7)
No	47 (27.3)	175 (55.6)	222 (45.6)	72 (33.5)	354 (69.0)	426 (58.5)	<0.001	119 (30.7)	529 (63.9)	648 (53.3)
Missing	0 (0.0)	3 (1.0)	3 (0.6)	7 (3.3)	26 (5.1)	33 (4.5)		7 (1.8)	29 (3.5)	36 (3.0)
**How often did you drink?**^3^										
Less than once a week	100 (80.0)	127 (92.7)	227 (86.6)	125 (91.9)	130 (97.7)	255 (94.8)	0.001	225 (86.2)	257 (95.2)	482 (90.8)
About once a week	14 (11.2)	5 (3.6)	19 (7.3)	4 (2.9)	0 (0.0)	4 (1.5)		18 (6.9)	5 (1.9)	23 (4.3)
More than once a week	11 (8.8)	4 (2.9)	15 (5.7)	6 (4.4)	2 (1.5)	8 (3.0)		17 (6.5)	6 (2.2)	23 (4.3)
Missing	0 (0.0)	1 (0.7)	1 (0.4)	1 (0.7)	1 (0.8)	2 (0.7)		1 (0.4)	2 (0.7)	3 (0.6)
**Ever smoked in past year**										
Yes	51 (29.7)	13 (4.1)	64 (13.1)	56 (26.0)	12 (2.3)	68 (9.3)		107 (27.6)	25 (3.0)	132 (10.9)
No	117 (68.0)	295 (93.7)	412 (84.6)	146 (67.9)	461 (89.9)	607 (83.4)	0.077	263 (68.0)	756 (91.3)	1019 (83.9)
Missing	4 (2.3)	7 (2.2)	11 (2.3)	13 (6.0)	40 (7.8)	53 (7.3)		17 (4.4)	47 (5.7)	64 (5.3)
**How many cigarettes per day have you smoked?**										
Less than 1–1 smoke occasionally	26 (51.0)	9 (69.2)	35 (54.7)	41 (73.2)	11 (91.7)	52 (76.5)	0.005	67 (62.6)	20 (80.0)	87 (65.9)
1–5 cigarettes per day	19 (37.3)	4 (30.8)	23 (35.9)	7 (12.5)	0 (0.0)	7 (10.3)		26 (24.3)	4 (16.0)	30 (22.7)
6–10 cigarettes per day	5 (9.8)	0 (0.0)	5 (7.8)	4 (7.1)	0 (0.0)	4 (5.9)		9 (8.4)	0 (0.0)	9 (6.8)
>10 cigarettes per day	1 (2.0)	0 (0.0)	1 (1.6)	3 (5.4)	0 (0.0)	3 (4.4)		4 (3.7)	0 (0.0)	4 (3.0)
Missing	0 (0.0)	0 (0.0)	0 (0.0)	1 (1.8)	0 (0.0)	2 (2.9)		1 (0.9)	1 (4.0)	2 (1.5)
**Ever taken Methamphetamine in past year**										
Yes	11 (6.4)	0 (0.0)	11 (2.3)	7 (3.3)	1 (0.2)	8 (1.1)		18 (4.7)	1 (0.1)	19 (1.6)
No	161 (93.6)	315 (100.0)	476 (97.7)	208 (96.7)	512 (99.8)	720 (98.9)	0.110	369 (95.3)	827 (99.9)	1196 (98.4)
**Ever used Marijuana in past year**										
Yes	14 (8.1)	0 (0.0)	14 (2.9)	20 (9.3)	4 (0.8)	24 (3.3)		34 (8.8)	4 (0.5)	38 (3.1)
No	158 (91.9)	315 (100.0)	473 (97.1)	195 (90.7)	509 (99.2)	704 (96.7)	0.679	353 (91.2)	824 (99.5)	1177 (96.9)
**Ever used Glue in past year**										
Yes	2 (1.2)	0 (0.0)	2 (0.4)	2 (0.9)	0 (0.0)	2 (0.3)		4 (1.0)	0 (0.0)	4 (0.3)
No	170 (98.8)	315 (100.0)	485 (99.6)	213 (99.1)	513 (100.0)	726 (99.7)	0.685	383 (99.0)	828 (100.0)	1211 (99.7)
**Ever used Ice drugs in past year**										
Yes	2 (1.2)	0 (0.0)	2 (0.4)	1 (0.9)	0 (0.0)	1 (0.1)		3 (0.8)	0 (0.0)	3 (0.2)
No	170 (98.8)	315 (100.0)	485 (99.6)	214 (99.5)	513 (100.0)	727 (99.9)	0.347	384 (99.2)	828 (100.0)	1212 (99.8)
**Ever used Heroin (non-injected) in past year**										
Yes	0 (0.0)	0 (0.0)	0 (0.0)	0 (0.0)	0 (0.0)	0 (0.0)		0 (0.0)	0 (0.0)	0 (0.0)
No	172 (100.0)	315 (100.0)	487 (100.0)	215 (100.0)	513 (100.0)	728 (100.0)	N/A	387 (100.0)	828 (100.0)	1215 (100.0)
**Ever used Injected any illegal drugs in past year**										
Yes	0 (0.0)	0 (0.0)	0 (0.0)	0 (0.0)	0 (0.0)	0 (0.0)		0 (0.0)	0 (0.0)	0 (0.0)
No	172 (100.0)	315 (100.0)	487 (100.0)	215 (100.0)	513 (100.0)	728 (100.0)	N/A	387 (100.0)	828 (100.0)	1215 (100.0)
**Ever used other illegal drugs in past year**										
Yes	0 (0.0)	0 (0.0)	0 (0.0)	0 (0.0)	0 (0.0)	0 (0.0)		0 (0.0)	0 (0.0)	0 (0.0)
No	172 (100.0)	315 (100.0)	487 (100.0)	215 (100.0)	513 (100.0)	728 (100.0)	N/A	387 (100.0)	828 (100.0)	1215 (100.0)

^1^ Ethnic minorities: Hmong, Karen, Akha, Lisu, Shan, Burmese, and Yunnanese

^2^ compared all lowland Thai and all ethnic minorities; *P* value from Chi-square test

*N/A* *(Non applicable due to insufficient number of outcomes in either of the level of the category)*

^3^ Percentage was calculated from those who ever drunk or smoked in the past year.

### Sexual behavior

As shown in Table 3, 51.1% of the respondents had a boy/girlfriend at the time of study, and 24.3% reported ever had sex with their current boy/girlfriend. Overall, 17.9% of the respondents (14.6% of female and 24.8% of male students) reported “ever had sex.” Among those who had ever had sex, 49% had only one partner, while 44.7% had two or more partners. Thirteen percent had their sexual debut before the age of 15. The first sex partner was a boy/girlfriend or other friend for 91% and a relative/neighbor/stranger for 5% (with 4% missing responses). Only one male respondent reported that he had his first act of sex with a sex worker.

**Table 3:** Sexual experience of high-school students in rural Chiang Mai, Thailand.

	Lowland Thai			Ethnic minorities^1^			*P* value^3^	Total		
	Males (N = 172)	Females (N = 315)	Total (N = 487)	Males (N = 215)	Females (N = 513)	Total (N = 728)		Males (N = 387)	Females (N = 828)	Total (N = 1215)
**Currently having boy/girlfriend**										
Yes	90 (52.3)	201 (63.8)	291 (59.9)	89 (41.4)	241 (47.0)	330 (45.3)	<0.001	179 (46.3)	442 (53.4)	621 (51.1)
No	82 (47.7)	113 (35.9)	195 (40.1)	126 (58.6)	271 (52.8)	397 (54.5)		208 (53.7)	384 (46.4)	592 (48.7)
Missing	0 (0.0)	1 (0.3)	1 (0.2)	0 (0.0)	1 (0.2)	1 (0.2)		0 (0.0)	2 (0.2)	2 (0.2)
**Ever had sex with current boy/girlfriend**										
Yes	29 (32.2)	72 (35.8)	101 (34.7)	18 (20.2)	32 (13.3)	50 (15.2)	<0.001	47 (26.3)	104 (23.5)	151 (24.3)
No	59 (65.6)	129 (64.2)	188 (64.6)	71 (79.8)	209 (86.7)	280 (84.8)		130 (72.6)	338 (76.5)	468 (75.4)
Missing	2 (2.2)	0 (0.0)	2 (0.7)	0 (0.0)	0 (0.0)	0 (0.0)		2 (1.1)	0 (0.0)	2 (0.3)
**Ever had sex**										
Yes	52 (30.2)	81 (25.7)	133 (27.3)	44 (20.5)	40 (7.8)	84 (11.5)	<0.001	96 (24.8)	121 (14.6)	217 (17.9)
No	120 (69.8)	234 (74.3)	354 (72.7)	171 (79.5)	473 (92.2)	644 (88.5)		291 (75.2)	707 (85.4)	998(82.1)
**Number of life time sexual partner**^4^										
≥ 2 partners	28 (53.8)	41 (50.6)	69 (51.9)	16 (36.4)	12 (30.0)	28 (33.3)	0.003	44 (45.8)	53 (43.8)	97 (44.7)
1 partner	16 (30.8)	38 (46.9)	54 (40.6)	26 (59.1)	27 (67.5)	53 (63.1)		42 (43.8)	65 (53.7)	107 (49.3)
Missing	8 (15.4)	2 (2.5)	10 (7.5)	2 (4.5)	1 (2.5)	3 (3.6)		10 (10.4)	3 (2.5)	13 (6.0)
**Sexual debut**^4^										
< 15 years	8 (15.4)	11 (13.6)	19 (14.3)	8 (18.2)	1 (2.5)	9 (10.7)	0.391	16 (16.7)	12 (9.9)	28 (12.9)
≥ 15 years	38 (73.1)	67 (82.7)	105 (78.9)	35 (79.5)	37 (92.5)	72 (85.7)		73 (76.0)	104 (86.0)	177 (81.6)
Missing	6 (11.5)	3 (3.7)	9 (6.8)	1 (2.3)	2 (5.0)	3 (3.6)		7 (7.3)	5 (4.1)	12 (5.5)
**First sex partner**^4^										
Boyfriend/girlfriend/other friend	40 (76.9)	79 (97.5)	119 (89.5)	39 (88.6)	39 (97.5)	78 (92.9)	0.335^F^	79 (82.3)	118 (97.5)	197 (90.8)
Relative/ Neighbor/ Stranger	7 (13.5)	1 (1.2)	8 (6.0)	3 (6.8)	0 (0.0)	3 (3.6)		10 (10.4)	1 (0.8)	11 (5.1)
Sex worker	0 (0.0)	0 (0.0)	0 (0.0)	1 (2.3)	0 (0.0)	1 (1.2)		1 (1.0)	0 (0.0)	1 (0.5)
Missing	5 (9.6)	1 (1.2)	6 (4.5)	1 (2.3)	1 (2.5)	2 (2.4)		6 (6.3)	2 (1.7)	8 (3.7)
**Use to avoid diseases/pregnancy (at first time had sexual)**^4^										
Withdrawal	12 (23.1)	25 (30.9)	37 (27.8)	6 (13.6)	7 (17.5)	13 (15.5)	0.019	18 (18.8)	32 (26.4)	50 (23.0)
Condom	27 (51.9)	31 (38.3)	58 (43.6)	31 (70.5)	25 (62.5)	56 (66.7)		58 (60.4)	56 (46.3)	114 (52.5)
Morning after pill	3 (5.8)	7 (8.6)	10 (7.5)	2 (4.5)	2 (5.0)	4 (4.8)		5 (5.2)	9 (7.4)	14 (6.5)
Traditional herbal medicine	4 (7.7)	14 (17.3)	18 (13.5)	4 (9.1)	4 (10.0)	8 (9.5)		8 (8.3)	18 (14.9)	26 (12.0)
Did not use any method	1 (1.9)	2 (2.5)	3 (2.3)	0 (0.0)	0 (0.0)	0 (0.0)		1 (1.0)	2 (1.7)	3 (1.4)
Missing	5 (9.6)	2 (2.5)	7 (5.3)	1 (2.3)	2 (5.0)	3 (3.6)		6 (6.3)	4 (3.3)	10 (4.6)
**History of sexually transmitted disease**^4^										
Yes	15 (28.8)	41 (50.6)	56 (42.1)	16 (36.4)	24 (60.0)	40 (47.6)	0.426	31 (32.3)	65 (53.7)	96 (44.2)
No	37 (71.2)	40 (49.4)	77 (57.9)	28 (63.6)	16 (40.0)	44 (52.4)		65 (67.7)	56 (46.3)	121(55.8)
**Consistent condom use**^4^										
Never	8 (15.4)	6 (7.4)	14 (10.5)	6 (13.6)	7 (17.5)	13 (15.5)	0.604	14 (14.6)	13 (10.7)	27 (12.4)
Occasionally	25 (48.1)	46 (56.8)	71 (53.4)	22 (50.0)	21 (52.5)	43 (51.2)		47 (49.0)	67 (55.4)	114 (52.5)
All of the time	10 (19.2)	20 (24.7)	30 (22.6)	10 (22.7)	9 (22.5)	19 (22.6)		20 (20.8)	29 (24.0)	49 (22.6)
Missing	9 (17.3)	9 (11.1)	18 (13.5)	6 (13.6)	3 (7.5)	9 (10.7)		15 (15.6)	12 (9.9)	27 (12.4)
**Use other method of birth control**^2^^,^^4^										
No	27 (51.9)	45 (55.6)	72 (54.1)	28 (63.6)	28 (70.0)	56 (66.7)	0.083	55 (57.3)	73 (60.3)	128 (59.0)
Yes	16 (30.8)	27 (33.3)	43 (32.3)	10 (22.7)	9 (22.5)	19 (22.6)		26 (27.1)	36 (29.8)	62 (28.6)
Missing	9 (17.3)	9 (11.1)	18 (13.5)	6 (13.6)	3 (7.5)	9 (10.7)		15 (15.6)	12 (9.9)	27 (12.4)
**Ever been pregnant or made someone pregnant**^5^										
Yes	1 (1.9)	6 (7.4)	7 (5.3)	0 (0.0)	0 (0.0)	0 (0.0)	0.047^F^	1 (1.0)	6 (5.0)	7 (3.2)
No	38 (73.1)	71 (87.7)	109 (82.0)	32 (72.7)	37 (92.5)	69 (82.1)		70 (72.9)	108 (89.3)	178 (820)
Missing	13 (25.0)	4 (4.9)	17 (12.8)	12 (27.3)	3 (7.5)	15 (17.9)		25 (26.0)	7 (5.8)	32 (14.7)

^1^ Ethnic minorities: Hmong, Karen, Akha, Lisu, Shan, Burmese, and Yunnanese

^2^ other method of birth control: Morning after pill, Injection, Intra-Uterine Device (IUD), Norplant

^3^ compared all lowland Thai and all ethnic minorities; *P* value from Chi-square test

^4^ data restricted to the subgroup of sexually active youth

^5^ data restricted to the subgroup of sexually active youth and had pregnant or made someone pregnant

^F^ Fisher’s exact test

*N/A* *(Non applicable due to insufficient number of outcomes in either of the level of the category)*

Compared to ethnic minority group respondents, lowland Thai respondents were more likely to have a boy/girlfriend at the time of the study (59.9% vs. 45.3%), to have had sex with their current boy/girlfriend (34.7% vs. 15.2%), to have ever had sex (27.3% vs. 11.5%), and to have had ≥ 2 lifetime sexual partners (51.9% vs. 33.3%).

### Condoms, contraception, sexually transmitted diseases, & pregnancy

Fifty-three percent of the study participants used condoms in order to avoid disease/pregnancy at their first sex, while the rest used withdrawal or other methods. When asked about the frequency of condom use since their initiation into sexual activity, only 22.6% reported using condoms all the time. On the other hand, 52.5% used them occasionally, and 12.4% never used them. Forty-four percent of the respondents reported ever having had either symptoms of STIs or had been diagnosed with STIs. Other methods of contraception (i.e. contraceptive pill, injections, intrauterine device, and Norplant) were used by 30% of female and 27% of male respondents. Five percent of female respondents (all were lowland Thai) had ever become pregnant, and half of those who ever had been pregnant ended with abortion. When compared with the lowland Thai and ethnic group, condom use during their first instance of sex was more frequently reported by ethnic minorities than by lowland Thai people (66.7% vs. 43.6%, p = 0.019) (see Table 3).

### Factors associated with having sexual intercourse

Table 4 displays bivariate associations with selected predictors with “ever having had sexual intercourse” respectively among the lowland Thai, ethnic minorities, and in the entire sample. Lowland Thai youth were more likely to report “ever having had sexual intercourse” compared to adolescents in the ethnic minority groups. The variables “ever having drunk alcohol in the past year”, “ever having smoked in the past year”, “ever having used methamphetamine in the past year”, “ever having used marijuana in the past year”, and “currently having boy/girlfriend” were significantly associated with “ever having had sexual intercourse” in both lowland Thai and ethnic minority groups.

**Table 4:** Bivariate analysis of factors associated with having sexual intercourse among high-school students in rural Chiang Mai, Thailand.

	Lowland Thai			Ethnic minorities^1^			Total		
	Yes (N = 133)	No (N = 354)	OR (CI)	Yes (N = 84)	No (N = 644)	OR (CI)	Yes (N = 217)	No (N = 998)	OR (CI)
**Ethnicity**									
Ethnic minorities							133 (61.3)	354 (35.5)	1
Lowland Thai							84 (38.7)	644 (64.5)	2.88 (2.12–3.89) ^†^
**Sex**									
Male	52 (39.1)	120 (33.9)	1	44 (52.4)	171 (26.6)	1	96 (44.2)	291 (29.2)	1
Female	81 (60.9)	234 (66.1)	0.79 (0.52–1.20)	40 (47.6)	473 (73.4)	0.32 (0.20–0.52) ^†^	121 (55.8)	707 (70.8)	0.51 (0.38–0.70) ^†^
**Age**									
16	5 (3.8)	46 (13.0)	1	9 (10.7)	97 (15.1)	1	14 (6.5)	143 (14.3)	1
17	91 (68.4)	245 (69.2)	3.41 (1.31–8.86) *	38 (45.2)	278 (43.2)	1.47 (0.68–3.15)	129 (59.4)	523 (52.4)	2.51 (1.40–4.50) **
18	34 (25.6)	56 (15.8)	5.58 (2.02–15.43) **	27 (32.1)	213 (33.1)	1.36 (0.61–3.01)	61 (28.1)	269 (27.0)	2.31 (1.25–4.28) **
19	3 (2.3)	7 (2.0)	3.94 (0.76–20.27)	10 (11.9)	56 (8.7)	1.92 (0.73–5.02)	13 (6.0)	63 (6.3)	2.10 (0.93–4.74) ^φ^
**Religion**									
Buddhism	129 (97.0)	338 (95.5)	1	61 (72.6)	365 (56.8)	1	190 (87.6)	703 (70.5)	1
Other	4 (3.0)	16 (4.5)	0.65 (0.21–1.99)	23 (27.4)	278 (43.2)	0.49 (0.29–0.82) **	27 (12.4)	294 (29.5)	0.34 (0.22–0.52) ^†^
**Living situation**									
Live with parents or relatives	118 (88.7)	283 (79.9)	1	48 (57.1)	329 (51.1)	1	166 (76.5)	612 (61.3)	1
Live with teachers	6 (4.5)	39 (11.0)	0.36 (0.15–0.89) *	25 (29.8)	249 (38.7)	0.68 (0.41–1.14)	31 (14.3)	288 (28.9)	0.39 (0.26–0.59) ^†^
Live with friends	1 (0.8)	4 (1.1)	0.60 (0.06–5.42)	6 (7.1)	26 (4.0)	1.58 (0.61–4.04)	7 (3.2)	30 (3.0)	0.86 (0.37–1.99)
Live alone	8 (6.0)	27 (7.6)	0.71 (0.31–1.61)	4 (4.8)	16 (2.5)	1.71 (0.55–5.34)	12 (5.5)	43 (4.3)	1.02 (0.53–1.99)
Live with other	0 (0.0)	0 (0.0)	N/A	1 (1.2)	24 (3.7)	0.28 (0.03–2.16)	1 (0.5)	24 (2.4)	0.15 (0.02–1.14) ^φ^
**Have access to mobile phone**									
I own one myself	126 (94.7)	317 (90.1)	1	65 (78.3)	450 (71.2)	1	191 (88.4)	767 (77.9)	1
I have access to one my family own	7 (5.3)	35 (9.9)	0.50 (0.21–1.16)	18 (21.7)	177 (28.0)	0.70 (0.40–1.22)	25 (11.6)	212 (21.5)	0.47 (0.30–0.73) **
No I do not have access to one at home	0 (0.0)	0 (0.0)	N/A	0 (0.0)	5 (0.8)	N/A	0 (0.0)	5 (0.5)	N/A
**Have access to internet**									
I own one myself	71 (54.6)	187 (54.7)	1	41 (49.4)	248 (41.5)	1	112 (52.6)	435 (46.3)	1
I have access to one my family own	8 (6.2)	25 (7.3)	0.84 (0.36–1.95)	12 (14.5)	83 (13.9)	0.87 (0.43–1.74)	20 (9.4)	108 (11.5)	0.71 (0.42–1.21)
No I do not have access to one at home	51 (39.2)	130 (38.0)	1.03 (0.67–1.57)	30 (36.1)	267 (44.6)	0.68 (0.41–1.12)	81 (38.0)	397 (42.2)	0.79 (0.57–1.08)
**Ever drunk alcohol in the past year**									
No	25 (18.8)	197 (56.1)	1	21 (25.6)	405 (66.1)	1	46 (21.4)	602 (62.4)	1
Yes	108 (81.2)	154 (43.9)	5.52 (3.40–8.96) ^†^	61 (74.4)	208 (33.9)	5.65 (3.35–9.54) ^†^	169 (78.6)	362 (37.6)	6.11 (4.30–8.67) ^†^
**Ever smoke in the past year**									
No	97 (75.2)	315 (90.8)	1	55 (67.9)	552 (92.9)	1	152 (72.4)	867 (92.1)	1
Yes	32 (24.8)	32 (9.2)	3.24 (1.89–5.57) ^†^	26 (32.1)	42 (7.1)	6.21 (3.54–10.90) ^†^	58 (27.6)	74 (7.9)	4.47 (3.04–6.56) ^†^
**Ever taken methamphetamine in the past year**									
No	124 (93.2)	352 (99.4)	1	82 (97.6)	638 (99.1)	1	206 (94.9)	990 (99.2)	1
Yes	9 (6.8)	2 (0.6)	12.77 (2.73–59.93) **	2 (2.4)	6 (0.9)	2.59 (0.51–13.06)	11 (5.1)	8 (0.8)	6.60 (2.62–16.63) ^†^
**Ever used marijuana in the past year**									
No	122 (91.7)	351 (99.2)	1	75 (89.3)	629 (97.7)	1	197 (90.8)	980 (98.2)	1
Yes	11 (8.3)	3 (0.8)	10.54 (2.89–38.44) ^†^	9 (10.7)	15 (2.3)	5.03 (2.12–11.89) ^†^	20 (9.2)	18 (1.8)	5.52 (2.87–10.64) ^†^
**Currently have a boyfriend/girlfriend**									
No	17 (12.9)	178 (50.3)	1	16 (19.3)	381 (59.2)	1	33 (15.3)	559 (56.0)	1
Yes	115 (87.1)	176 (49.7)	6.84 (3.94–11.86) ^†^	67 (80.7)	263 (40.8)	6.06 (3.43–10.70) ^†^	182 (84.7)	439 (44.0)	7.02 (4.74–10.38) ^†^

^1^ Ethnic minorities: Hmong, Karen, Akha, Lisu, Shan, Burmese, and Yunnanese

* *P* value < 0.05

** *P* value < 0.01

^*†*^*P* value <0.001

^φ^ *P* value <0.10

OR odds ratio; CI: confidence interval

In the adjusted models (Table 5), the increased odds of Thai adolescents to report “ever having had sexual intercourse” remained statistically significant [Adjusted odds ratio (AOR), 1.61; CI, 1.06–2.45; *P*< 0.01)]. In the entire sample, factors associated with “ever having had sexual intercourse” included being aged ≥ 17 years (AOR, 2.69; CI, 1.34–5.43; *P* = 0.002) and 18 years (AOR, 3.62; CI, 1.72–7.61; *P =* 0.000), “ever having drunk alcohol in the past year” (AOR, 3.70; CI, 2.45–5.58; *P* = 0.000), “ever having smoked in the past year” (AOR, 1.83; CI, 1.06–3.14; *P* = 0.001), and “currently having boy/girlfriend” (AOR, 6.89; CI, 4.43–70.73; *P* = 0.000).

**Table 5:** Multivariate analysis of factors associated with having sexual intercourse among high-school students in rural Chiang Mai, Thailand.

Independent variable	Lowland Thai (N = 487)		Ethnic minorities^1^ (N = 728)		Total (N = 1215)	
	Adjusted odds ratio (95% CI)	*P* value^#^	Adjusted odds ratio (95% CI)	*P* value^#^	Adjusted odds ratio (95% CI)	*P* value^#^
**Ethnicity**						
Ethnic minorities					1	
Lowland Thai					1.61 (1.06–2.45)	0.001
**Sex**						
Male	1		1		1	
Female	1.42 (0.78–2.59)	0.285	0.43 (0.23–0.80)	0.001	0.82 (0.54–1.24)	0.001
**Age**						
16	1		1		1	
17	5.69 (1.65–19.66)	0.012	1.56 (0.63–3.88)	0.319	2.69 (1.34–5.43)	0.002
18	10.31 (2.72–39.05)	<0.001	1.70 (0.66–4.40)	0.440	3.62 (1.72–7.61)	<0.001
19	1.31 (0.13–13.53)	0.101	2.52 (0.76–8.36)	0.181	2.63 (0.95–7.29)	0.072
**Religion**						
Buddhism	1		1		1	
Other	0.70 (0.17–2.91)	0.457	0.52 (0.28–0.98)	0.016	0.61 (0.35–1.05)	0.073
**Living situation**						
Live with parent(s) or relatives	1		1		1	
Live with teacher(s)	0.63 (0.20-.98)	0.072	0.69 (0.36–1.31)	0.151	0.67 (0.39–1.14)	0.060
Live with friends	2.58 (0.18–37.67)	0.649	2.30 (0.70–7.52)	0.338	1.82 (0.65–5.09)	0.726
Live alone	0.64 (0.22–1.90)	0.413	2.14 (057–8.03)	0.353	0.84 (0.36–1.94)	0.933
Live with other	N/A		0.46 (0.05–4.46)	0.225	0.56 (0.06–5.23)	0.067
**Have access to mobile phone**						
I own one myself	1		1		1	
I have access to one my family own	0.90 (0.31–2.67)	0.108	1.12 (0.55–2.26)	0.211	1.02 (0.58–1.79)	0.059
No, I do not have access to one at home	N/A		N/A		N/A	
**Have access to internet**						
I own one myself	1		1		1	
I have access to one my family own	0.51 (0.17–157)	0.690	1.11 (0.46–2.70)	0.703	1.02 (0.53–1.99)	0.215
No, I do not have access to one at home	0.99 (0.58–1.68)	0.880	0.88 (0.47–1.64)	0.131	0.96 (0.65–1.42)	0.150
**Ever drunk alcohol in past year**						
No	1		1		1	
Yes	4.94 (2.78–8.77)	<0.001	2.42 (1.28–4.58)	0.001	3.70 (2.45–5.58)	<0.001
**Ever smoked in past year**						
No	1		1		1	
Yes	1.25 (0.57–2.75)	0.053	3.06 (1.40–6.72)	0.001	1.83 (1.06–3.14)	0.001
**Ever taken methamphetamine in past year**						
No	1		1		1	
Yes	12.25 (1.41–106.70)	0.001	0.39 (0.05–3.03)	0.248	2.16 (0.62–7.50)	<0.001
**Ever used marijuana in past year**						
No	1		1		1	
Yes	2.37 (0.39–14.62)	0.679	1.27 (0.41–3.99)	0.226	1.50 (0.59–3.81)	0.746
**Currently having boy/girlfriend**						
No	1		1		1	
Yes	7.68 (3.99–14.78)	<0.001	7.01 (3.69–13.28)	<0.001	6.89 (4.43–10.73)	<0.001

^1^ Ethnic minorities: Hmong, Karen, Akha, Lisu, Shan, Burmese, and Yunnanese

^#^ *P* value from multiple logistic regression

CI confidence interval

Significant factors common to members of both lowland Thai and ethnic minority groups were “ever having drunk alcohol in the past year” (lowland Thai: AOR, 4.94; CI, 2.78–8.77; *P* = 0.000, ethnic minorities: AOR, 2.42; CI, 1.28–4.58; *P* = 0.001) and currently having boy/girlfriend (lowland Thai: AOR, 7.68; CI, 3.99–14.78; *P* = 0.000, ethnic minorities: AOR, 7.01; CI, 3.69–13.28; *P* = 0.000). Specifically, for lowland Thai high-school students, being aged 17 (AOR, 5.69; CI, 1.65–19.66; *P* = 0.012) and 18 years (AOR, 10.31; CI, 2.72–39.05; *P =* 0.000), and “ever having taken methamphetamine in the past year” (AOR, 12.25; CI, 1.41–106.70; *P* = 0.001) were associated with increased odds of “ever having had sexual intercourse.” Among the respondents from ethnic minority groups, being female (AOR, 0.43; CI, 0.23–0.80; *P* = 0.001) and practicing religions other than Buddhism (AOR, 0.52; CI, 0.28–0.98; *P* = 0.016) were associated with decreased odds of ever had sexual intercourse, while “ever had smoked in the past year” was associated with increased odds of ever had sexual intercourse (AOR, 3.06; CI, 1.40–6.72; *P* = 0.001) (see Table 4).

## Discussion

We found that adolescents attending high school in rural Chiang Mai engage in a number of risky patterns of sexual behavior, and that significant differences exist between lowland adolescent Thai youth and adolescent youth from ethnic minorities with respect to their risky patterns of sexual behaviors, including sexual intercourse experience; condom use; boyfriend or girlfriend relationship status; and number of lifetime sexual partners.

Overall, 17.9% of adolescents reported “ever having had sexual intercourse”. Among these adolescents, lowland Thai adolescents were more likely to report “ever having had sexual intercourse” compared to adolescents from ethnic minority groups. Risky patterns of sexual behavior were more prevalent among lowland Thai youth than among the youth of the minority groups in the Chiang Mai area. A higher proportion of lowland Thai youth had at least 2 lifetime sexual partners, and currently had a boyfriend or a girlfriend. A lower proportion of them used condoms during their first incident of sexual intercourse—as compared to adolescents from ethnic minority groups.

The observed difference between the two groups can possibly be explained—first—by the fact that lowland Thai and ethnic minority groups may value educational opportunity differently. Although access to primary education has substantially increased among ethnic minority groups, access to higher education is still limited among these groups, especially for those without documentary proof of nationality and for those without financial support. Therefore, access to high school among adolescents from ethnic minority groups may be regarded as a valuable opportunity, and as an incentive for a strong commitment to school among adolescents in this group. This may to a certain degree have a protective effect against engaging in risky sexual behaviors. Secondly, lowland adolescent Thai youth, compared to their ethnic minority counterparts, may be relatively more exposed to globalization. The impacts of this phenomenon on the sexual norms of young people have been extensively documented [22–26]. For example, in our study, a significantly higher proportion of the lowland adolescent Thai youth owned a mobile phone and had personal access to the Internet, as compared to ethnic minority groups. Globalization has accelerated the adoption of a new international culture and norms through enhanced interconnectivity between people and also through exposure to modern media including magazines, movies, and the Internet.

A recent study in Thailand conducted in an urban setting has documented changes in the sexual norms of Thai youth. These have been characterized by a decline in the age of sexual debut; a larger number of lifetime sexual partners, and a greater acceptance of adolescent premarital sex [5]. In the same study, younger generation of Thai had more positive attitudes toward women carrying condoms [5]. In light of our study, it is possible that similar changes are also occurring in the rural areas of northern Thailand. Although our data did not demonstrate a significant association between having access to mobile phone or internet and “ever having sexual intercourse”, more exploration using qualitative study design might be warranted to learn more about effects of globalization on youths’ life style, attitude, and sexual orientation. These changes are probably happening specifically among lowland Thai adolescents with little protective traditional and religious safeguards. Furthermore, the strong attachment of ethnic minority groups to their customs, traditional values, and religions certainly shape their sexual norms, and consequently render behaviors such as premarital sex which was inappropriate in the community.

In our study, religion had no effect on the sexual experience among the lowland Thai youth. However, the effect of practicing any religion other than Buddhism (mostly Christianity) was associated with lower odds of “ever had sex” among adolescents from ethnic minority groups. The fact that a very limited proportion (3.9%) of lowland adolescent Thai practiced religion other than Buddhism have precluded a reliable judgment on the protective effect of these religions on risky sexual behaviors. However, a previous study in urban Chiang Mai found similar results on the effect of religion on patterns of sexual risk behaviors. Many other studies in other settings have also demonstrated the effectiveness of faith-based HIV prevention interventions [27–29]. More research is needed to explore the effects, and to explore the pathways through which religion influence sexual morality of adolescents in rural northern Thailand.

It is important to signal that although patterns of sexual risk behaviors were much more prevalent among lowland adolescent Thai than among their counterparts from ethnic minorities, both groups of adolescents remain largely vulnerable to HIV infection and other STIs. For instance, among the sexually active adolescents attending high school in rural Chiang Mai, a significantly low proportion (22.6%) used condoms consistently, and nearly half of them had 2 or more sexual partners. The same pattern of behaviors was also noted in our recent study among young people attending non-formal education centers in urban Chiang Mai [11]; underscoring the particular vulnerability of young Thai to HIV and other STIs. Although many HIV prevention and reproductive health initiatives and campaigns have been conducted in Chiang Mai [30], consistent use of condoms among adolescents continues to be unacceptably low [4]. There is need for continued research to understand the dynamics of sexual behavior patterns, in order to inform the design and implementation of interventions that are tailored to the needs of adolescents, and address correlates of their risky sexual behaviors.

The finding that very few adolescent males in our study had their first sexual experience with commercial sex workers, as was common in past years, has been observed in a number of studies. The fear of contracting HIV infection through commercial sex workers whose links with HIV infection were frequently reported during the early HIV prevention campaigns has led young males to rely on their girlfriends for sexual needs [7, 31–33].

We found that alcohol use was invariably associated with increased likelihood of “ever had sexual intercourse” among both lowland Thai adolescents and adolescents from ethnic minority groups. The association of alcohol use with sexual risk behaviors is well documented in the literature [34–38]. As previously reported in a study in Thailand, alcohol consumption is perceived by youth as allowing people to more easily break social and traditional norms which served as a brake to engaging in risky sexual behaviors [36]. Another study of vocational students in northern Thailand showed that at any given age, sexual initiation was associated with alcohol consumption [35].

In the study results, in addition to alcohol, tobacco smoking was associated with having sexual intercourse among both lowland adolescent Thai and adolescents from ethnic minorities. It is therefore crucial for interventions promoting safe sexual behaviors among adolescents in rural Chiang Mai, and in Thailand in general, to incorporate information addressing the negative consequence of substance use.

This study has the merit of being one the first studies to document differences in risky sexual behaviors between lowland adolescent Thai and adolescents from ethnic minority groups attending high school. More importantly, our study has policy implications—as it shows that adolescents in rural areas of Thailand are at large vulnerable to HIV infection and STIs. Ministries of Health and Education, NGOs, and other important stakeholders should energize their efforts to make sure that interventions and health campaigns reach out to the rural adolescents.

There are several limitations, however, to this research. Firstly, our study is limited for its use of a convenient sampling scheme which reduces the possibilities of generalizing our findings. Although it is clear that the results of this study do, to a large extent, represent the situation of adolescents in rural Chiang Mai, it is not clear to what extent these findings can be generalized to other adolescents in other areas of Thailand. Also, our sample included only adolescents aged 16–19 years. Adolescents younger or older than those included in our age bracket may have different behavioral patterns. Secondly, we cannot draw any causal inferences, due to the cross-sectional design nature of our study. There is risk of social desirability bias—given the sensitivity inherent to sexual health topics. Lastly,

In summary, we have found that although important differences in risky patterns of sexual behavior do exist between lowland Thai adolescents and adolescents from ethnic minority groups, both groups remain largely vulnerable to HIV and STIs. We have been able to identify a number of factors associated with sexual intercourse experience including alcohol consumption; smoking; methamphetamine usage; age; and having boy/girlfriend. Sex education programs in schools are absolutely required. Furthermore, these programs need to be standardized in order to raise the awareness of young people about risky behavior patterns and how to avoid risky situations. Such interventions should, however, consider the broader contextual and structural landscape within which the adolescents in urban Chiang Mai live.

## Supporting Information

S1 Dataset S1_dataset. (SAV)

S1 Questionnaire S1_Questionnaire—Thai. (DOCX)

S2 Questionnaire S2_Questionnaire—English. (DOCX)
