# Supplementary material for: Patterns of Sexual Behavior in Lowland Thai Youth and Ethnic Minorities Attending High School in Rural Chiang Mai, Thailand
Source: PLoS One. 2016 Dec 1;11(12):e0165866. doi: 10.1371/journal.pone.0165866 (PMC5132398; doi:10.1371/journal.pone.0165866)
Supplement: S1 Questionnaire — (DOCX) [file pone.0165866.s002.docx]

เลขที่แบบสอบถาม _ _ _ _ [สำหรับผู้วิจัย]

| **แบบสอบถาม**  **โครงการวิจัย: การป้องกันเอดส์และการตั้งครรภ์ไม่พึงประสงค์ในเยาวชนกลุ่ม**  **ชาติพันธุ์ ในจังหวัดเชียงใหม่**  **วิธีการตอบคำถาม:**  กาเครื่องหมาย ถูก 🗸 ลงบนเส้นประ __ ที่ต่อจากคำตอบที่เลือก ยกตัวอย่างเช่น 🗸  หรือเติมตัวเลขลงบนเส้นประ __ __ ยกตัวอย่างเช่น **1 8** |
| --- |

**ตอนที่ 1: ข้อมูลทางด้านสังคมประชากร**

วันที่ตอบแบบสอบถาม (วันที่-เดือน-พ.ศ.) _ _ - _ _ - _ _

1.1 ท่านเป็นเพศชายหรือเพศหญิง?

ชาย (1) _ หญิง (2) _

1.2 เมื่อวันครบรอบวันเกิดที่ผ่านมา ท่านมีอายุเท่าไหร่? _ _ ปี

1.3 ท่านนับถือศาสนาอะไร?

พุทธ (1) _ คริสต์ (2) _ อิสลาม (3) _

ไม่นับถือศาสนาใด (4) _ อื่น ๆ (โปรดระบุ)___________________ (5) _

1.4 บรรพบุรุษของท่านสืบเชื้อสายจากชนกลุ่มใด?

จีน (1) _ พม่า (2) _ ไทใหญ่ (3) _

ไทยภูเขา (4) (ระบุ)_________ ไทยพื้นราบ (5) _ อื่นๆ (ระบุ)____________(6) _

1.5 ปัจจุบันท่านพักอาศัยอยู่กับใคร?

อาศัยอยู่กับพ่อแม่/ญาติ เช่น พี่ ป้า น้า ยาย (1) _ อาศัยอยู่กับครู (2) _

อาศัยอยู่กับเพื่อนในห้องเช่าหรือบ้านเช่า (3) _ อาศัยอยู่คนเดียวในห้องเช่าหรือบ้านเช่า (4) _

อาศัยอยู่กับผู้อื่น (โปรดระบุ) ___________ (5) _

**ตอนที่ 2: ข้อมูลเกี่ยวกับวิถีชีวิต**

2.1 ขณะนี้ท่านหรือคนในครอบครัวได้เป็นเจ้าของสิ่งต่อไปหรือไม่?

|  | **ฉันและครอบครัวไม่มีสิ่งนี้** | **ใช่, ฉันมีเป็นของตนเอง** | **ฉันไม่มีเป็นของตนเอง**  **แต่ครอบครัวของฉันมี** | **ฉันมีเป็นของตนเอง และครอบครัวของฉันก็มี** |
| --- | --- | --- | --- | --- |
| โทรศัพท์มือถือ |  |  |  |  |
| อินเตอร์เน็ต (ในมือถือ/เครื่องคอมพิวเตอร์) |  |  |  |  |

2.2 ในระยะ 1 ปี ที่ผ่านมา ท่านได้ดื่มเครื่องดื่มที่มีแอลกอฮอล์ หรือไม่?

ดื่ม (1) _ ไม่ดื่ม (2) _ **ถ้าไม่ดื่ม ให้ข้ามไปข้อ 2.3**

2.2.1 ถ้าดื่ม ท่านดื่มเครื่องดื่มที่มีแอลกอฮอล์บ่อยแค่ไหน?

ดื่มนาน ๆ ครั้ง (1) _ ดื่มประมาณ 1ครั้งต่อสัปดาห์ (2) _

ดื่มมากกว่า 1 ครั้งต่อสัปดาห์ (3) _

2.2.2 โดยเฉลี่ยแล้วท่านดื่มเครื่องดื่มที่มีแอลกอฮอล์ครั้งละกี่แก้ว?

1-2 แก้ว (1) _ 3-5 แก้ว (2) _ มากกว่า 5 แก้ว (3) _

2.3 ในระยะ 1 ปี ที่ผ่านมา ท่านได้สูบบุหรี่หรือไม่?

สูบ (1) _ ไม่สูบ (2) _ **ถ้าไม่สูบ ข้ามไปข้อ 2.4**

2.3.1 ถ้าสูบ โดยเฉลี่ยแล้วท่านสูบบุหรี่บ่อยแค่ไหน?

สูบนาน ๆ ครั้ง (1) _ 1-5 มวนต่อวัน (2) _

6-10 มวนต่อวัน (3) _ มากกว่า 10 มวนต่อวัน (4) _

2.4 ในระยะ 1 ปี ที่ผ่านมา ท่านได้เสพสิ่งต่อไปนี้หรือไม่ อย่างไร?

|  | **เสพประมาณ** | **เสพนาน ๆ ครั้ง** | **ไม่ได้เสพสิ่งนี้** |
| --- | --- | --- | --- |
| ยาบ้า | __ __ ครั้ง/เดือน | __ | __ |
| กัญชา | __ __ ครั้ง/เดือน | __ | __ |
| สารระเหย | __ __ ครั้ง/เดือน | __ | __ |
| ยาไอซ์ | __ __ ครั้ง/เดือน | __ | __ |
| เฮโรอีน (สูบ) | __ __ ครั้ง/เดือน | __ | __ |
| ฉีดสารเสพติดเข้าเส้นเลือด | __ __ ครั้ง/เดือน | __ | __ |
| อื่น ๆ (ระบุ) ___________ | __ __ ครั้ง/เดือน | __ | __ |

**ตอนที่ 3: ข้อมูลเกี่ยวกับความรักความสัมพันธ์**

3.1 ในระยะ 1 ปี ที่ผ่านมา ท่านได้ใช้เวลาว่างส่วนใหญ่อยู่กับใคร? **(ตอบได้หลายข้อเลือก)**

เพื่อน (1) _ แฟน (2) _ มารดา (3) _

บิดา (4) _ พี่น้อง (5) _ ญาติ (6) _

อยู่กับตัวเอง (7) _

3.2 ขณะนี้ท่านมีแฟนหรือไม่?

มี (1) _ ไม่มี (2) _ **ถ้าไม่มี ให้ข้ามไปตอนที่ 4**

3.2.1 แฟนของท่านอายุเท่าไหร่? __ __ ปี

3.2.2 แฟนของท่านเป็นเพศชายหรือเพศหญิง?

ผู้ชาย (1) _ ผู้หญิง (2) _

3.2.3 ท่านวางแผนจะแต่งงาน / มีความสัมพันธ์แบบถาวร กับแฟนคนนี้หรือไม่?

ใช่ (1) _ ไม่ใช่ (2) _ ยังไม่แน่ใจ (3) _

3.2.4 ท่านเคยมีเพศสัมพันธ์กับแฟนคนนี้หรือไม่?

เคย (1) _ ไม่เคย (2) _

**ตอนที่ 4: ข้อมูลเกี่ยวกับเพศสัมพันธ์**

4.1 ท่านเคยมีเพศสัมพันธ์หรือไม่?

*[เพศสัมพันธ์ หมายถึง อวัยวะเพศของคนใดคนหนึ่งสอดใส่เข้าไปในช่องคลอดหรือทวารหนักของอีกคนหนึ่ง]*

เคย (1) _

ไม่เคย (2) _

**ถ้าไม่เคยมีเพศสัมพันธ์ ให้ข้ามไปตอบในตอนที่ 7**

4.2 ในชีวิตที่ผ่านมาท่านมีเคยเพศสัมพันธ์กับคนทั้งหมดกี่คน _ _ คน

4.3 ท่านอายุเท่าไหร่เมื่อมีเพศสัมพันธ์**ครั้งแรก**? อายุ _ _ ปี

4.3.1 เพศสัมพันธ์ครั้งแรกเกิดขึ้นที่ไหน?

ที่พักของฉัน (1) _ ที่พักของคู่นอน (2) _

ที่พักของเพื่อน (3) _ โรงแรมหรือโมเต็ล (4) _

ในรถยนต์ (5) _ ในสถานบริการทางเพศ (6) _

ในที่โล่งแจ้ง (7)_ อื่น ๆ (โปรดระบุ) ___________ (8) _

4.3.2 คู่นอนคนแรกของท่านเป็นใคร?

แฟน (1) _ เพื่อน (2) _

ญาติ (3) _ คนรู้จัก (4) _

คนแปลกหน้า (5) _ ผู้ขายบริการทางเพศ (6) _

อื่น ๆ (โปรดระบุ) _______________ (7) _

4.3.3 คู่นอนคนแรกของท่านเป็นเพศชายหรือเพศหญิง?

ผู้ชาย (1) _ ผู้หญิง (2) _

4.3.4 ในเพศสัมพันธ์ครั้งแรก ท่าน/คู่นอนได้ใช้วิธีการป้องกันโรค/การตั้งครรภ์หรือไม่ อย่างไร?

หลั่งภายนอก (1) _ ถุงยางอนามัย (2) _

ใช้ยาคุมกำเนิดฉุกเฉิน (3) _ ยาฆ่าเชื้อหลังร่วม (4) _

ใช้ยาพื้นบ้าน/ยาสมุนไพร (5) _ ไม่ได้ใช้วิธีใด ๆ (6) _

อื่น ๆ (โปรดระบุ) _______________ (7) _

4.4 ท่านมีเพศสัมพันธ์**ครั้งสุดท้าย**เมื่อไหร่**?**

1-2 วัน ที่ผ่านมา (1) _ ภายใน 1 สัปดาห์ ที่ผ่านมา (2) _

ภายใน 1 เดือน ที่ผ่านมา (3) _ ภายใน 3 เดือน ที่ผ่านมา (4) _

ภายใน 1 ปี ที่ผ่านมา (5) _ มากกว่า 1 ปี ที่ผ่านมา (6) _

4.4.1 ในครั้งสุดท้ายนั้น คู่นอนของท่านเป็นใคร?

แฟน (1) _ เพื่อน (2) _ ญาติ (3) _

คนรู้จัก (4) _ คนแปลกหน้า (5) _ ผู้ขายบริการทางเพศ (6) _

อื่น ๆ (โปรดระบุ) _______________ (7) _

4.4.2 คู่นอนของท่านในตอนนั้นเป็นชายหรือหญิง?

ผู้ชาย (1) _ ผู้หญิง (2) _

4.4.3 ในครั้งนั้น ท่าน/คู่นอนได้ใช้วิธีการป้องกันโรค / การตั้งครรภ์หรือไม่ อย่างไร? หลั่งภายนอก (1) _ ถุงยางอนามัย (2) _

ยาเม็ดคุมกำเนิดชนิดแผง (3) _ ใช้ยาคุมกำเนิดฉุกเฉิน (4) _

ยาฆ่าเชื้อหลังร่วม (5) _ ใช้ยาพื้นบ้าน/ยาสมุนไพร (6) _

ไม่ได้ใช้วิธีใด ๆ (7) _ อื่น ๆ (โปรดระบุ) _______________ (8)_

**ตอนที่ 5: *โรคติดต่อทางเพศสัมพันธ์***

5.1 ท่านเคยมีอาการ หรือเคยเป็นโรคต่าง ๆ ต่อไปนี้หรือไม่?

|  | **เคย** | **ไม่เคย** | **ไม่แน่ใจ / ไม่ทราบ** |
| --- | --- | --- | --- |
| แผลหรือตุ่มที่อวัยวะสืบพันธุ์ | **__** | **__** | **__** |
| ปวด/แสบ/ขัด เวลาปัสสาวะ | **__** | **__** | **__** |
| คันบริเวณอวัยวะสืบพันธุ์ | **__** | **__** | **__** |
| มีสารคัดหลั่งที่ผิดปกติ (เช่นมีหนอง หรือมีมูกสีเหลืองหรือเขียว) ออกจากอวัยวะสืบพันธุ์ | **__** | **__** | **__** |
| ช่องคลอด/ปากมดลูกอักเสบ (เฉพาะเพศหญิง) | **__** | **__** | **__** |
| ปีกมดลูกอักเสบ (เฉพาะเพศหญิง) | **__** | **__** | **__** |
| ท่อปัสสาวะอักเสบ | **__** | **__** | **__** |
| หนองใน | **__** | **__** | **__** |
| ซิฟิลิส | **__** | **__** | **__** |
| หนองในเทียม (คลาไมเดีย) | **__** | **__** | **__** |
| เริม | **__** | **__** | **__** |
| ติดเชื้อ เอช ไอ วี | **__** | **__** | **__** |
| อื่น ๆ (โปรดระบุ)___________ ___________ | **__** | **__** | **__** |

**ถ้าตอบไม่เคย หรืไม่แน่ใจ/ไม่ทราบ ให้ข้ามไปตอบตอนที่ 6**

5.2 ถ้าเคย ครั้งสุดท้ายที่ผ่านมา ท่านทำอย่างไร?

ไปสถานีอนามัยหรือโรงพยาบาลรัฐบาล (1) _ ไปคลินิกหรือโรงพยาบาลเอกชน (2) _

ไปร้านขายยา (3) _ หายาจากที่อื่นมารับประทานเอง (4) _

ไม่ทำอย่างไร (5) _ อื่น ๆ (โปรดระบุ) ____________ (6) _

**ตอนที่ 6: *การคุมกำเนิดและการตั้งครรภ์***

6.1 ตั้งแต่ท่านมีเพศสัมพันธ์ครั้งแรกจนถึงปัจจุบัน ท่านและคู่นอนใช้วิธีการคุมกำเนิดต่อไปนี้หรือไม่อย่างไร?

|  | **ใช้ตลอดเวลา** | **ใช้เป็นส่วนใหญ่** | **ใช้เป็นบางโอกาส** | **ไม่เคยใช้เลย** |
| --- | --- | --- | --- | --- |
| หลั่งภายนอก | __ | __ | __ | __ |
| นับระยะปลอดภัย | __ | __ | __ | __ |
| ใช้ถุงยางอนามัย | __ | __ | __ | __ |
| ยาเม็ดคุมกำเนิด | __ | __ | __ | __ |
| ยาคุมกำเนิดฉุกเฉิน | __ | __ | __ | __ |
| ยาฉีดคุมกำเนิด | __ | __ | __ | __ |
| ห่วงอนามัย | __ | __ | __ | __ |
| ยาฝังคุมกำเนิด | __ | __ | __ | __ |
| ยาพื้นบ้านหรือยาสมุนไพร | __ | __ | __ | __ |
| อื่น ๆ โปรดระบุ_________ | __ | __ | __ | __ |

6.2 ท่านเคยตั้งครรภ์หรือทำให้คู่นอนของท่านตั้งครรภ์หรือไม่?

เคย (1) _ ไม่เคย (2) _ **ถ้าไม่เคย จบการตอบแบบสอบถาม**

6.2.1 ถ้าเคย เคยกี่ครั้ง? _ ครั้ง

6.2.2 ผลของการตั้งครรภ์เป็นอย่างไรบ้าง? (ตอบได้หลายข้อเลือก)

ทำแท้ง (1) _ แท้งเอง (2) _ คลอด (3) _

กำลังตั้งครรภ์อยู่ (4) _ อื่น ๆ (โปรดระบุ) _________________ (5) __

**ผู้ที่มีประสบการณ์ทางเพศแล้ว จบการตอบแบบสอบถาม**

**ขอขอบคุณท่านเป็นอย่างยิ่ง ในการให้ความร่วมมือตอบแบบสอบถาม**

**ตอนที่ 7: สำหรับผู้ที่ไม่มีประสบการณ์ทางเพศเท่านั้น**

ผู้คนอาจมีเหตุผลที่หลากหายในการไม่มีเพศสัมพันธ์ คำถามต่อไปนี้จะถามท่านเกี่ยวกับความคิดเห็นของตัวท่านเกี่ยวกับเรื่องนี้

7.1 เหตุผลที่ท่านไม่มีเพศสัมพันธ์

|  | **ใช่** | **ไม่ใช่** | **ไม่แน่ใจ** |
| --- | --- | --- | --- |
| ฉันรู้สึกยังไม่พร้อมที่จะมีเพศสัมพันธ์ | __ | __ | __ |
| ฉันยังหาโอกาสไม่ได้ | __ | __ | __ |
| ฉันคิดว่าการมีเพศสัมพันธ์ก่อนแต่งงานเป็นสิ่งที่ไม่ถูกต้อง | __ | __ | __ |
| ฉันกลัวท้อง / กลัวทำคนอื่นท้อง | __ | __ | __ |
| ฉันกลัวติดเอดส์ / กลัวโรคติดต่อทางเพศสัมพันธ์ | __ | __ | __ |
| ฉันแคร์ความรู้สึกของพ่อแม่ | __ | __ | __ |
| ฉันกลัวคนที่สถานศึกษาจะตำหนิหรือนินทา | __ | __ | __ |
| อื่น ๆ (โปรดระบุ)____________________ | __ | __ | __ |

7.2 ในอนาคต ท่านคิดว่าจะวางแผนในการมีเพศสัมพันธ์เมื่อใด?

ฉันจะรอจนถึงตอนแต่งงาน (1) _ ฉันจะรอจนถึงมีการหมั้นหมายกันก่อน (2) _

ฉันจะรอจนถึงเมื่อพบคนที่ฉันรัก (3) _ ฉันจะรอจนถึงเมื่อมีโอกาส (4) _

ฉันวางแผนที่จะไม่มีเพศสัมพันธ์กับใครเลย (5) _ ยังไม่แน่ใจในอนาคต (6) _

อื่น ๆ (โปรดระบุ) ________________________________ (7) _

7.3 ท่านรู้สึกถูกกดดันที่จะต้องมีเพศสัมพันธ์หรือไม่

ใช่ ฉันถูกกดดันมาก (1) _ ใช่ แต่ฉันถูกกดดันเล็กน้อยเท่านั้น (2) _

ฉันไม่ถูกกดดันเลย (3) _ **ถ้าไม่ถูกกดดันเลย จบการตอบแบบสอบถาม**

7.3.1 ถ้าใช่ ท่านถูกกดดันจากใคร (เลือกได้มากกว่า 1 ข้อเลือก)

แฟน (1) _ เพื่อนเพศเดียวกัน (2) _

เพื่อนต่างเพศ (3) _ สื่อต่าง ๆ (4) _

อื่น ๆ (โปรดระบุ) ___________ (5) _

**ขอขอบคุณท่านเป็นอย่างยิ่ง ในการให้ความร่วมมือตอบแบบสอบถาม**
