# Supplementary material for: Patterns of Sexual Behavior in Lowland Thai Youth and Ethnic Minorities Attending High School in Rural Chiang Mai, Thailand
Source: PLoS One. 2016 Dec 1;11(12):e0165866. doi: 10.1371/journal.pone.0165866 (PMC5132398; doi:10.1371/journal.pone.0165866)
Supplement: S2 Questionnaire — (DOCX) [file pone.0165866.s003.docx]

Study ID number _ _ _ _ [For official use only]

| **Questionnaire survey**  **Study program: Prevention of AIDS and unwanted pregnancy in young ethnic minority people in Chiang Mai**  **How to answer:**  Just put a tick in the space __ next to the appropriate answer like this **√**.  Or write in a number on a line __ __ like this **1 8**. |
| --- |

**Section 1: Socio-demographic information**

Interview date (dd-mm-yy Buddhist Era.) _ _-_ _-_ _

1.1 Were you born male or female?

Male (1) _

Female (2) _

1.2 What was your age last birthday?

_ _ Years

1.3 What is your religion?

Buddhism (1) _ Christianity (2) _

Islam (3) _ No religion (4) _

Other (Specify) ___________ (5) _

1.4 What is the race/ethnicity of your family?

Chinese (1) _ Burmese (2) _

Shan or Tai Yai (3) _ Hill tribes (specify) ________(4) _

Thai (from Northern or other regions) (5) _ Other (specify) ___________ (6) _

1.5 What is your current living situation?

Live with parent(s) or relative(s) (1) _

Live with teachers (2) _

Share rented room or house with friend(s) (3) _

Live alone in rented room or house (4) _

Other (specify) ____________________ (5) _

**Section 2: Lifestyle information**

2.1 Do you currently have access at home to the following items?

|  | No, I do not have access to one at home | Yes, I own one myself | I don’t own one myself, but I have access to one my family owns | I own one myself, and I have access to one my family owns |
| --- | --- | --- | --- | --- |
| Mobile phone |  |  |  |  |
| Internet (from mobile phone or computer) |  |  |  |  |

2.2 Have you ever drunk alcohol (e.g. beer, whisky, wine) during the past year?

Yes (1) _

No (2) _

***If no skip to Q 2.3***

2.2.1 If yes, how often did you drink?

Less than once a week (1) _

About once a week (2) _

More than once a week (3) _

2.2.2 On average, how many drinks did you usually have at one time? (One drink = one beer, one whisky-soda, one glass of wine, etc.)

1-2 drinks (1) _

3-5 drinks (2) _

More than 5 drinks (3) _

2.3 Have you ever smoked cigarettes during the past year?

Yes (1) _

No (2) _

***If no skip to Q* 2.4**

2.3.1 If yes, on average, how many cigarettes per day have you smoked?

Less than 1 – I smoke occasionally (1) _

1-5 cigarettes (2) _

6-10 cigarettes (3) _

More than 10 cigarettes (4) _

2.4 During the past year, how often have you taken the following drugs?

|  | **Regularly*,**  **with approximately** | **Occasionally** | **Never** |
| --- | --- | --- | --- |
| Methamphetamines *(yaa baa)* | __ __ time(s)/month |  |  |
| Marijuana (*ganja*) | __ __ time(s)/month |  |  |
| Glue | __ __ time(s)/month |  |  |
| Ice drugs (*yaa ice*) | __ __ time(s)/month |  |  |
| Heroin (non-injected) | __ __ time(s)/month |  |  |
| Injected any illegal drugs | __ __ time(s)/month |  |  |
| Other (specify) __________ | __ __ time(s)/month |  |  |

* Regularly means you take at least once a month on that type of drug.

**Section 3: Relationship information**

3.1 In the past year, who did you mainly spend your free time with? (You may give more than one answer)

Friends (1) _

Boyfriend/girlfriend (2) _

Mother (3) _

Father (4) _

Siblings (5) _

Other relatives (6) _

Myself, on my own (7) _

3.2 Do you currently have a boyfriend/girlfriend?

Yes (1) _

No (2) _

***If no, skip to Section 4***

3.2.1 How old is your boyfriend/girlfriend?

_ _ years

3.2.2 Are they male or female?

Male (1) _

Female (2) _

3.2.3 Do you plan to get married/have a permanent relationship with him/her?

Yes (1) _

No (2) _

Not sure (3) _

3.2.4 Have you had sex with your boyfriend/girlfriend?

Yes (1) _

No (2) _

**Section 4: Sexual experience**

4.1 Have you ever had sexual intercourse?

*[Sexual intercourse means that one person’s penis was inserted in the vagina or anus of another person.]*

Yes (1) _

No (2) _

***If never had sexual intercourse, skip to Section 7***

4.2 How many different people have you ever had sexual intercourse with?

__ __ Persons

4.3 How old were you when you had sexual intercourse for the **first time**?

Age _ _ years old

4.3.1 Where did it happen?

My room/house (1) _ Partner’s room/house (2) _

Friend’s room/house (3) _ Motel or hotel (4) _

Car (5) _ Brothel (6) _

Outdoors (7) _ Other (specify) ___________ (8) _

4.3.2 Who was your first sex partner?

Boyfriend/girlfriend (1) _

Other friend (2) _

Relative (3) _

Neighbour (4) _

Stranger (5) _

Sex worker (6) _

Other (specify) _______________ (7) _

4.3.3 Was your partner a male or a female?

Male (1) _

Female (2) _

4.3.4 **The first time** you had sexual intercourse, which method did you and your partner use to avoid diseases/pregnancy?

Withdrawal of penis before ejaculation (1) _

Condom (2) _

Morning after pill (3) _

Germ killers (tablets or gel) (4) _

Traditional herbal medicines (5) _

We did not use any method (6) _

Other (specify) _______________ (7) _

4.4 When was the **last time** you had sexual contact/intercourse?

Within the last two days (1) _

Within the last week (2) _

Within the last month (3) _

Within the last 3 months (4) _

Within the last year (5) _

More than a year ago (6) _

4.4.1 Who was your last sex partner?

Boyfriend/girlfriend (1) _ Other friend (2) _

Relative (3) _ Neighbour (4) _

Stranger (5) _ Sex worker (6) _

Other (specify) _______________ (7) _

4.4.2 Was your partner on that occasion a male or a female?

Male (1) _

Female (2) _

4.4.3 **The last time** you had sexual intercourse, which method did you and your partner use to avoid diseases/pregnancy?

Withdrawal of penis before ejaculation (1) _

Condom (2) _

Oral pill (3) _

Morning after pill (4) _

Germ killers (tablets or gel) (5) _

Traditional herbal medicines (6) _

We did not use any method (7) _

Other (specify) _______________ (8) _

**Section 5: Sexually transmitted diseases**

5.1 Have you ever experienced any of following signs / symptoms / diseases.

|  | **Yes** | **No** | **Not sure / Don’t know** |
| --- | --- | --- | --- |
| Ulcer or sore in genital area |  |  |  |
| Painful/burning sensation when urinating |  |  |  |
| Itching around the opening of the penis/vagina |  |  |  |
| Abnormal genital discharge (a white, yellow, or green discharge from penis/vagina) |  |  |  |
| Vaginitis/Cervicitis (females only) |  |  |  |
| Pelvic Inflammatory Disease (females only) |  |  |  |
| Urethritis |  |  |  |
| Gonorrheae |  |  |  |
| Syphilis |  |  |  |
| Chlamydia |  |  |  |
| Genital herpes |  |  |  |
| HIV infection |  |  |  |
| Other sexually transmitted disease (specify)___________ |  |  |  |

***If No or Not sure/Don’t know to ALL items, skip to Section 6***

5.2 If yes to any item listed, last time when you had one of the above symptoms, what did you do?

Visited doctor at public hospital/clinic (1) _

Visited doctor at private hospital/clinic (2) _

Went to pharmacy asking for advice and to buy drugs (3) _

Bought drugs from somewhere else to treat myself (4) _

Did nothing (5) _

Other (specify) _______________ (6) _

**Section 6: Birth control and pregnancy**

6.1 Since you first had sexual intercourse, how regularly have you and your partner(s) used each of the following methods of birth control?

|  | **All of the time** | **Most of the time** | **Occasionally** | **Never** |
| --- | --- | --- | --- | --- |
| Withdrawal of penis before ejaculation |  |  |  |  |
| Periodic abstinence |  |  |  |  |
| Condom |  |  |  |  |
| Oral pill |  |  |  |  |
| Morning after pill |  |  |  |  |
| Injection |  |  |  |  |
| Intra-Uterine Device (IUD) |  |  |  |  |
| Norplant |  |  |  |  |
| Traditional herbal remedies |  |  |  |  |
| Other, specify___________ |  |  |  |  |

6.2 Have you ever been pregnant, or made a sexual partner of yours pregnant?

Yes (1) _

No (2) _

***If no, end of questionnaire***

6.2.1 If yes, how many times?

_ Time (s)

6.2.2 What were the outcomes of the pregnancies? (You may answer more than one choice)

Abortion (1) _

Miscarriage (2) _

Delivery (3) _

Still pregnant (4) _

Other, specify___________ (5) _

***For those who have had sexual experience, end of questionnaire.***

***Thank you very much for taking time to answer the questionnaire.***

**Section 7: FOR THOSE WHO HAVE NEVER HAD SEXUAL INTERCOURSE**

7.1 Reasons why you may not have had sexual intercourse:

|  | **Yes** | **No** | **Not sure** |
| --- | --- | --- | --- |
| I don't feel ready to have sex. |  |  |  |
| I have not had the opportunity to have sex. |  |  |  |
| I think that sex before marriage is wrong**.** |  |  |  |
| I am afraid of getting pregnant or causing a pregnancy if I have sex**.** |  |  |  |
| I am afraid of getting HIV/AIDS or another sexually transmitted infection if I have sex. |  |  |  |
| I don’t have sex because I care about my parents’ feelings. |  |  |  |
| I am afraid of negative reactions from school if I have sex. |  |  |  |
| Other (specify) __________________________ |  |  |  |

7.2 Which of these statements best describes your plans regarding having sex?

I plan to wait until marriage (1) _

I plan to wait until I am engaged to be married (2) _

I plan to wait until I find someone I love (3) _

I plan to have sexual intercourse whenever an opportunity comes along (4) _

I plan not to have sexual intercourse with anyone (5) _

I am not sure about whether or when I will have sex in the future (6) _

Other (specify) _______________ (7) _

7.3 Do you feel any pressure from others to have sexual intercourse?

A great deal (1) _

A little (2) _

None (3) _

***If none, end of questionnaire***

7.3.1 From whom do you feel pressure? (You may answer more than one choice)

Boyfriend/girlfriend (1) _

Same sex friends (2) _

Friends of the opposite sex (3) _

Mass media (4) _

Other (specify) _______________ (5) _

***Thank you very much for taking time to answer the questionnaire.***
